# Practical Notes on Diagnosis and Treatment

**Published:** 1910-06-25

**Authors:** 


### Practical Notes on Diagnosis and Treatment.

### Piles.

When piles are present the endeavour should be made to get the bowels to act regularly before going to bed rather than in the morning.

### Sulphate of Soda.

Glauber's salt has a stimulating action on the liver, but should not be used as an habitual purgative on account of its irritative action on the stomach. It should be mixed with bicarbonate of soda to lessen this effect. It is superior to any other drug in preventing the increased formation of uric acid.—*Dr. Huchard.*

### Syringing the Ear.

The dangers incurred in syringing out the middle ear in the presence of a chronic discharge are very great indeed. All watery fluids encourage bacterial growth and enhance what is the main object to prevent and avoid. Syringing is always liable to have the effect of driving sepsis further afield. I never syringe on any pretext, and entirely disapprove of syringing the ear in any way whatever in the presence of a discharge. Wiping out the ear with twisted wool is all that is necessary and advisable to entrust to the patient.—*Mr. W. Stuart-Low.*

### Intestinal Action in the New-born.

Meconium, owing to its physical qualities, is admirably designed to lubricate the mucous surface of the intestines and to offer a convenient medium of resistance to the peristaltic movements. It is important in stimulating the first efforts of the intestines and in founding the persistence of a correct habit. If the mild and gentle stimulus of meconium be replaced by a purgative dose of castor oil, all subsequent reaction will be materially modified. I know of no series of purgative medicines which are responsible for so much constipation as the single dose of castor oil which clears out meconium from the bowel of the new-born infant.—*Dr. Eric Pritchard.*

### Hæmorrhage.

The application of a tampon soaked in a solution of gelatine will be found useful in producing clotting. The clot is firm and lasting, much more coherent than that produced by perchloride of iron. The solution used should be about 10 per cent. gelatine in normal saline.

### Scarlatinal Otitis.

The radical mastoid operation is preferable in scarlatinal cases; in catarrhal cases the operation limited to the mastoid side may be best. For the after-treatment of the radical mastoid operation, packing with gauze is used for a week only; thereafter irrigation, followed by instillation of a saturated solution of boric acid in alcohol.—*Dr. Knyvett Gordon.*

### Dyspnœa in Pneumonia.

Although the breathing is short and rapid, and the patient appears to be the subject of considerable respiratory distress, he will often not acknowledge that he feels embarrassed in his breathing. It is otherwise if there is a swollen or catarrhal condition of the bronchial mucous membrane. This creates a mechanical obstruction to the breathing, throws more strain upon the right side of the heart, and adds considerably to the gravity of the prognosis.—*Sir Thomas Oliver.*

### Gastrostaxis or Ulcer?

The symptoms in a case of gastric ulcer do not subside so quickly as in one of gastrostaxis. In the latter a few days' rest and low feeding are followed by a rapid amelioration of all the symptoms. Hæmorrhage from a gastric ulcer implies the presence of active ulceration, and other signs will also be found—*e.g.* rigidity, tenderness, and localised pain. For two or three days the treatment will be the same in both diseases, but if the diagnosis of gastrostaxis seems justified the strength and quantity of the food are to be rapidly increased.—*Dr. G. A. Sutherland.*

